# Restructuring the Healthcare System to Protect Healthcare Personnel Amidst the COVID-19 Pandemic

**DOI:** 10.3389/fpubh.2020.588203

**Published:** 2020-12-11

**Authors:** Mona Duggal, Neha Dahiya, Ankita Kankaria, Manav Chaudhary, Damodar Bachani

**Affiliations:** ^1^Post Graduate Institute of Medical Education and Research (PGIMER), Chandigarh, India; ^2^All India Institute of Medical Sciences, Bathinda (AIIMS Bathinda), Bhatinda, India; ^3^University of British Columbia, Vancouver, BC, Canada; ^4^NCD, Ministry of Health & Family Welfare, New Delhi, India

**Keywords:** restructuring of health system, COVID-19, health care professionals, public health system, elderly

## Introduction

COVID-19 infection caused by SARS-CoV2 has been declared a pandemic by the World Health Organization. Reports from China and France have shown that older age is a prognosticator of severity and mortality (1, 2). The number of elderly COVID-19 cases and the increased death rates among them compared to the younger population are surfacing across the world. The odds of hospitalization and the requirement of ICU facilities for the elderly are very high, which further adds burdens to the already compromised system in India where 0.55 beds are available per 1,000 of the population (3). ICU care is also very low, which aggravates the situation (3). Data from other countries have shown that even though 20% of cases are elderly people, they account for 79% of deaths, since associated comorbidities like diabetes, hypertension, respiratory diseases, which are common in the older population, fan the flames (4–6). A model-based analysis from China demonstrated a compelling age gradient in the case of fatality ratio 0.32 in <60 years vs. 6.4% in >60 years and up to 13.4% in >80. Analogously, the hospitalization rate in infected individuals also upsurges with age (7). A study done from China analyzed data from 27 countries and highlighted age as the most important predictor for the odds of surviving from COVID-19 disease (8).

In Wuhan, more than 3,300, and in Italy 4,800, front line medical staff were infected^1^. In China, out of the total overall deaths, 4.4% were health care workers and the median age was 55 years (9). Likewise in the USA, where 9,282 cases were reported among health workers and the median age was 42 years and 74.5% were female^2^. In Indonesia, 115 doctors had died because of COVID-19 as of September 2020 (10). The rate of infection among doctors in India is very high in comparison to other countries. As of late September 2020, 2,238 doctors have been infected and around 380 have died due to coronavirus, with 75% of them above the age of 50 years (11, 12). In the UK, mortality was higher among Black and Asian doctors (13). This vulnerability varies across ages and states. Across the world, the majority of the healthcare workforce is above 50 years of age^1^ and they are at high risk of being infected, owing to their nature of work (14–16).

A single surgery or procedure could risk infecting multiple healthcare staff at the same time (17). Those who get infected may not develop symptoms themselves but can transmit the infection to colleagues or patients because of interaction in enclosed spaces, creating a causal nexus (18). Infected healthcare staff often go into quarantine or require hospitalization. As the number of beds and staff is already low, this shortage further strains the healthcare system and it is not easy to train new staff in a short period (19). Lack of testing facilities, shortage, and low-quality PPE has made the situation worse (20). Although the Indian government has relaxed leaves rules for staff above 50 (21), there are still not been any clear policies or guidelines for health staff above 50 years of age on protecting them from infection and other complications due to COVID-19. Prevention from infection does not only require masks and PPE, it also calls for measures and the restructuring of administration, engineering, and academia (22–24).

Public health care services, which are the lifeline of any country, need to be rejuvenated. In the current pandemic, effective public healthcare system models like Bhilwara, South Korea, and Kerala were able to control COVID-19 due to the implementation of intensive outreach based public health measures like contact tracing, case identification, home-based screening, home quarantine, and the restriction of movement (25). It has been observed that during this pandemic situation, most COVID-19 cases are being treated in public hospitals (26). Therefore, to enhance the scalability, sustainability, and build the resilience of public health systems in similar future unprecedented events, the gross domestic product needs to take a quantum jump from 1.15 to 4–5% and create robust public health systems and achieve universal health coverage (27).

Keeping the current situation in mind, the restructuring of health systems is needed and it may be prudent to recommend that only a younger, fitter, and robust health care force are at the forefront of care until more promising prevention strategies, such as a vaccine, have been developed. Another crucial approach is to reverse quarantine and urge senior healthcare staff to stay at home and provide guidance virtually, to limit their exposure to the virus (28, 29). Junior doctors such as post-graduate residents and interns should be allowed to run frontline health services and develop a deeper skillset (30). For instance, the operation theaters and emergencies could be managed by junior staff under the leadership of more senior staff. Seniors should be encouraged to work in non-clinical areas where the risk of infection is low. In this context, virtual rounds that take information from residents could be the new normal (31).

Periodic surveillance of all the health care workers for early detection and treatment of the virus is required. This will also help break the chain of transmission. Stanford tried weekly testing of staff (32). Regular webinars on stress management for healthcare professionals working in this high-stress environment may also be required. Instead of seeing the current situation as a challenge, it is time to take the opportunity to transition to a digitalized health system, removing implementation barriers, and investing in telehealth, which will bear fruit in the long term as a way of extending reach and impact, and simultaneously helping to accomplish Sustainable Development Goal 3 (33).

Every hospital should have a dedicated disaster committee or pandemic preparedness taskforce. A human resource management committee focusing on the duty schedule could provide support to family members of health workers. SMART roster policies of staff in COVID-19 wards (1 week of duty with 2 weeks of isolation) and the same individuals during isolation could support frontline teams virtually using technology. The deployment of a younger healthcare workforce including junior doctors and paramedics in outreach services in the community will decrease the burdens on resources such as PPE, beds, and ventilators and the risk of contagion in tertiary care. Strengthening the capacity and skills of medical and other frontline sectors is crucial in the current panic situation, and could be done virtually by senior health care workers (34). Telemedicine is needed to accelerate the curve of quality education and access to services whilst concomitantly flattening the curve of the epidemic (35).

Health care workers have greater exposure to severe disease patients. Surgeries and other aerosol-producing procedures like intubation, endoscopy, and resuscitation, etc. put them at higher risk of contracting the virus (36). The occupational hazard risks are not fully understood but generate the need for a tool to stratify healthcare workers at higher risks. Hence there is an urgent need to develop risk scores for deciding roles and duties. Such scoring should be based on age, presence of comorbidities (Heart disease/lung disease/diabetes/kidney disease/weak immune system), pregnancy, disability, training status, and family support, etc. (8, 37). The formulation of proper guidelines and checklists to control errors will be useful. Regular training audits and mock drills should be part of the system. Regular simulation modeling techniques should be used and will help in predicting the outcome of each measure.

The biomedical waste management, laundry, and housecleaning departments must be engaged in all infection control meetings (38). In Intensive care units, preprocedural briefings and post-procedural debriefings should be made mandatory. Patient zoning and flow should be managed strategically. It has been demonstrated that clinical triage tools to cohort and isolate the virus, potentially reducing the chances of hospital-acquired COVID-19 infection (39). The establishment of a separate triage building that would serve the purpose of efficient triage of patients should also be considered (40). The whole hospital can be divided into separate suspected COVID-19 zoned building and a non-COVID-19 building. A patient who does not fit any criteria of suspected COVID should only be referred from the triage building to the Non-COVID-19 building and the rest should be treated in the suspected COVID-19 building. Crowd control measures should be implemented. Cohorts of health staff that are divided into two sections reduce the risk of cross-infection of staff working in the high risk areas compared to staff working in the low risk area (39). The placement of staff in high-risk buildings should be assessed based on age, presence of comorbidity, social and familial circumstances (for instance if they are a single parent, etc.) (41). These personal circumstances may indicate that they should not be posted in high-risk buildings as strict infection control measures are imperative (42). Other engineering measures include social distancing, providing good ventilation in OPDs, and creating sheet barriers between doctors and patients (43). Periodic disinfection of the hospital is another engineering measure that requires consideration.

## Conclusion

Vertical Expansion of the healthcare system demands high funds and resources, whereas the horizontal integration and restructuring of the system seems to be the most appropriate, cost-effective, and sustainable approach. A multifaceted approach to redesigning the health system coupled with the integration of digital health will enable us to combat the current COVID-19 pandemic.

## Author Contributions

ND contributed to the conceptualization and manuscript writing. AK and MC helped in critical revision and manuscript writing. DB and MD contributed to the conceptualization, critical revision, and final approval of the version to be published. All authors contributed to the article and approved the submitted version.

## Conflict of Interest

The authors declare that the research was conducted in the absence of any commercial or financial relationships that could be construed as a potential conflict of interest.

## Footnotes

^1^https://www.theguardian.com/world/2020/apr/16/doctors-nurses-porters-volunteers-the-uk-health-workers-who-have-died-from-covid-19.

^2^https://www.cdc.gov/mmwr/volumes/69/wr/mm6915e6.htm.
